# PDIA3/ERp57 promotes a matrix-rich secretome that stimulates fibroblast adhesion through CCN2

**DOI:** 10.1152/ajpcell.00258.2021

**Published:** 2022-02-23

**Authors:** Andrew L. Hellewell, Kate J. Heesom, Mark A. Jepson, Josephine C. Adams

**Affiliations:** ^1^School of Biochemistry, University of Bristol, Bristol, United Kingdom; ^2^Faculty of Life Sciences, University of Bristol, Bristol, United Kingdom

**Keywords:** cell adhesion, extracellular matrix, interactome, secretome, thrombospondin-1

## Abstract

The matricellular glycoprotein thrombospondin-1 (TSP1) has complex roles in the extracellular matrix (ECM) and at cell surfaces, but relatively little is known about its intracellular associations prior to secretion. To search for novel intracellular interactions of TSP1 in situ, we carried out a biotin ligase-based TSP1 interactome screen and identified protein disulfide isomerase A3 (PDIA3/ERp57) as a novel candidate binding protein. In validation, TSP1 and PDIA3 were established to bind in vitro and to colocalize in the endoplasmic reticulum of human dermal fibroblasts (HDF). Loss of PDIA3 function, either by pharmacological inhibition in HDF or in *Pdia3^−/−^* mouse embryo fibroblasts (*Pdia3^−/−^* MEFs), led to alterations in the composition of cell-derived extracellular matrix, involving changed abundance of fibronectin and TSP1, was correlated with reduced cell spreading, altered organization of F-actin, and reduced focal adhesions. These cellular phenotypes of *Pdia3^−/−^* MEFs were normalized by exposure to conditioned medium (WTCM) or extracellular matrix (WTECM) from wild-type (WT)-MEFs. Rescue depended on PDIA3 activity in WT-MEFs and was not prevented by immunodepletion of fibronectin. Heparin-binding proteins in WTCM were found to be necessary for rescue. Comparative quantitative tandem-mass-tag proteomics and functional assays on the heparin-binding secretomes of WT-MEFs and *Pdia3^−/−^* MEFs identified multiple ECM and growth factor proteins to be downregulated in the CM of *Pdia3^−/−^* MEFs. Of these, cell communication network 2 (CCN2) was identified to be necessary for the adhesion-promoting activity of WTCM on *Pdia3^−/−^* MEFs and to bind TSP1. Thus, PDIA3 coordinates fibroblast production of an ECM-rich, proadhesive microenvironment, with implications for PDIA3 as a translational target.

## INTRODUCTION

The extracellular matrix (ECM) is a fundamental component of the microenvironment of animal cells that directs cell phenotype in physiological and pathological contexts throughout life. The activities of ECM proteins are typically mediated by molecular interactions at the cell surface, in the pericellular milieu and in the ECM; however, there is also growing evidence for intracellular activities of ECM proteins (1–4). In view of the multiplicity of roles of ECM proteins and that many ECM proteins have tissue-specific expression profiles, there is great interest to manipulate ECM composition for cell culture, bioengineering, or clinical applications. However, targeting of individual ECM proteins is complex, due to their abundance, large size and multidomain nature, and multiplicity of binding interactions.

Biosynthesis of ECM proteins typically involves many posttranslational modification steps and can include oligomerization. After cotranslational entry through the translocon to the lumen of the endoplasmic reticulum (ER), chaperones and enzymes ensure correct folding, posttranslational modification, and trafficking of ECM proteins before secretion. For example, lysine and proline residues in fibrillar collagens are hydroxylated by ER-resident lysyl and prolyl hydrolyases, respectively (5), to promote thermal stability of collagen triple-helix assembly. Export of collagen I (6), collagen IIα1, and matrilins (7) from the ER to the Golgi apparatus depends on the coat protein II complex (COPII) export factor. In addition, protein prodomains and additional ER-resident chaperones prevent protein-protein interactions that could trigger unwanted ECM assembly within the secretory pathway (8).

Mechanisms of collagen assembly and trafficking are a long-standing focus of ECM research; however, knowledge on the folding and trafficking of other ECM proteins is relatively sparse. These processes are of fundamental importance and potential translational value given the molecular complexity of tissue ECMs. Here, we have focused on thrombospondin-1 (TSP1), a member of the TSP family of multidomain, calcium-binding glycoproteins. TSPs act as matricellular proteins at cell surfaces and within the ECM to modulate cellular activities including cell-ECM interactions, growth factor signaling pathways, cell motility, and angiogenesis, among others (9–12). Mammals encode five TSPs, the trimeric TSP1 and TSP2 and the pentameric TSP3, TSP4, and TSP5 (TSP5 is also known as cartilage oligomeric matrix protein or COMP). Several TSPs are associated with human genetic diseases: single nucleotide polymorphisms in TSP1 and TSP4 are associated with increased risk of cardiovascular disease (13) and mutations in TSP5 are associated with pseudoachondroplasia (PSACH) (14, 15). In the chondrocytes of PSACH patients, mutant TSP5 accumulates in the ER along with several TSP5-binding ECM proteins, leading to enlargement of ER cisternae and, ultimately, premature cell death and weakening of cartilage ECM (16–19).

Much research has focused on extracellular interactions of TSPs, yet knowledge of their intracellular interactions within the secretory pathway is limited. For TSP1, appropriate folding and assembly in the ER of rat thyroid epithelial cells requires a complex of four chaperones: binding immunoglobulin protein (BiP), grp94, grp170, and ERp72 (20). The processing and transport of wild-type TSP5 in chondrocytes requires the ER chaperones calreticulin, protein disulfide isomerase (PDI/PDIA1), and grp94, whereas BiP appears to associate preferentially with mutant TSP5 (21). ER-located TSPs are also implicated in responses of cells to ER stress: for example, TSP4 overexpression in mouse cardiac tissue or cardiomyocytes triggers a protective ER stress response through its interaction with the ER-luminal domain of activating transcription factor 6α (atf6α) (22, 23).

In view of the many pathophysiological roles of TSP1 and the limited knowledge of its interactions before secretion, we set out to examine the intracellular interactome of TSP1 by the biotin ligase proximity-dependent ligation (BioID) method (24). BioID involves fusion of a protein of interest to a modified version of *Escherichia coli* biotin ligase bifunctional ligase/repressor (BirA) that biotinylates proximal amines promiscuously (24). In the presence of biotin, proteins in close proximity are biotinylated and can be isolated on avidin-beads and identified by mass spectrometry. Building on discovery of a novel interaction between TSP1 and protein disulfide isomerase A3 (PDIA3, also known as ERp57), we identify intracellular PDIA3 to control an ECM-rich and proadhesive secretome of functional significance in fibroblasts.

## MATERIALS AND METHODS

### Cell Culture

For a list of antibodies, see Table 1. Chemicals were from Sigma unless otherwise stated. *Helix pomatia* agglutinin (HPA) lectin-Alexa Fluor 647 conjugate (ThermoFisher Scientific, Cat. No. L32454) was used as a marker for the Golgi apparatus (25). Protein disulfide isomerase inhibitor 16F16 (26) was from Sigma (Cat. No. SML0021). Recombinant human TGF-β1 (Cat. No. 240-B) was from R&D. Recombinant human PDIA3/ERp57 was from Abcam (Cat. No. ab92937). Recombinant human PDI/PDIA1 was from Enzo (Cat. No. ENZ-51024). Native human platelet thrombospondin-1 was from Cell Sciences Inc. (Cat. No. CSL19832A). Recombinant human CCN2 was from R&D (Cat. No. 9190-CC-050). Wild-type (WT) and *Pdia3^−/−^* mouse embryonic fibroblasts (MEFs) were a kind gift from Marek Michelak, University of Alberta (27). MEFs were used between *passages 4* and *30* and were cultured either in DMEM (Sigma, Cat. No. D6429) containing 10% fetal calf serum (FCS; Sigma, Cat. No. F7524) or defined fibroblast growth medium (PromoCell, Cat. No. C-23010) containing 50 µg/mL l-ascorbic acid (Sigma, Cat. No. A4544). COS7 cells were cultured in DMEM containing 10% FCS. Normal human dermal fibroblasts (HDF) from juvenile foreskin (PromoCell, Cat. No. C-12300) were used between *passages 4* and *12* and cultured in fibroblast growth medium (PromoCell, Cat. No. C-23010) containing 50 µg/mL l-ascorbic acid. All cells were maintained in a humidified incubator with 5% CO_2_ at 37°C.

**Table 1.** List of antibodies used

Target	Species	Clonality	Conjugate	Source	Cat. No.	Dilution
Actin, α-smooth muscle	Mouse	Monoclonal (1A4)		Sigma	A2547	1/1,000 (IB)
CTGF/CCN2	Rabbit	Polyclonal		Abcam	ab6992	1/500 (IB)
Fibronectin	Rabbit	Polyclonal		Sigma	F3648	1/600 (IB), 1/100 (IF)
Fibronectin (ED-A)	Mouse	Monoclonal (IST9)		Abcam	ab6328	1/500 (IB)
GAPDH	Mouse	Monoclonal (mAbcam 9484)		Abcam	ab9484	1/600 (IB)
HA	Rabbit	Polyclonal		Santacruz	sc-805	1/2,000 (IB) 1/100 (IF)
KDEL	Mouse	Monoclonal (10C3)		Enzo	ADI-SPA-827-D	1/500 (IF)
KDEL	Rabbit	Polyclonal		Abcam	ab176333	1/500 (IF)
Mouse IgG	Goat	Polyclonal	HRP	Licor	926-80010	1/50,000 (IB)
Mouse IgG	Goat	Polyclonal	AF-488	Life Technologies	A-11001	1/500 (IF)
PDIA1	Rabbit	Monoclonal (C81H6)		Cell Signalling Technology	3501	1/2,000 (IB)
PDIA3/ERp57	Mouse	Monoclonal (MaP.Erp57)		Abcam	ab13506	1/1,000 (IB), 1/100 (IF)
PDIA3/ERp57	Rabbit	Polyclonal		Abcam	ab137567	1/600 (IB), 1/100 (IF)
Rabbit IgG	Goat	Polyclonal	HRP	Licor	926-80011	1/100,000 (IB)
Rabbit IgG	Sheep	Polyclonal	TRITC	ThermoFisher	A16177	1/200 (IF)
TSP1	Mouse	Monoclonal (C9)		Santacruz	sc-393503	1/200 (IB), 1/50 (IF)
TSP1	Mouse	Monoclonal (A6.1)		Santacruz	sc-59887	1/200 (IB), 1/50 (IF)
Vimentin	Rabbit	Monoclonal (EPR3776)		Abcam	ab92547	1/2,000 (IF)
Vinculin	Mouse	Monoclonal (VIN-11-5)		Sigma	VIN-11-5	1/200 (IB)

AF, Alexa Fluor; HRP, horseradish peroxidase; IB, immunoblot; IF, immunofluorescence; TRITC, tetramethylrhodamine isothiocyanate.

### Plasmids and Transfection

pcDNA3.1 MCS-BirA(R118G)-HA plasmid (24) was obtained from Addgene (36047). TSP1.V5 was as described (28). TSP1.BirA-HA construct was generated by PCR of *Homo sapiens* TSP1 cDNA with custom oligonucleotide primers 800 F: 5′GACTCTCGAGAAAGGACAACACCGTGCCC3′, and 801 R: 5′GTACTCTAGACTATGCGTAATCCGGTACATC3′ (synthesized by Sigma and provided desalted). The BirA-HA PCR product (993 bp) and the pcDNA3.1-TSP1.V5 plasmid were each digested with XhoI (NEB, Cat. No. R0146S) and XbaI (NEB, Cat. No. R0145S) and the plasmid treated with Antarctic phosphatase (NEB, Cat. No. M0289S) for 1 h. Reaction products were gel-purified (MinElute Gel Extraction Kit. Qiagen, Cat. No. 28606), the expected DNAs purified, and then ligated with Quick ligase (NEB, Cat. No. M2200S), transformed into XL-1-Blue supercompetent *E. coli* (Agilent, Cat. No. 200236), and colonies grown on LB-ampicillin plates. Plasmid DNA extracted from single colonies (QIAprep Spin Miniprep Kit. Qiagen, Cat. No. 27106) was digested with XhoI and XbaI restriction endonucleases to identify correctly ligated plasmid clones. From several clones with DNA inserts of the expected size, midiprep DNA (HiSpeed Plasmid Midi Kit, Qiagen, Cat. No. 12643) was purified and sent for DNA sequencing (Eurofins) to validate the entire sequence of the cDNA insert on one strand. COS7 cells (8 × 10^5^) were transfected with 2.5 µg of plasmid using Polyfect transfection reagent (Qiagen, Cat. No. 301107) according to the manufacturer’s instructions and cultured for 24 h, unless otherwise stated.

### Cell Fractionation and Immunoblotting

For extraction of total cell lysate (TCL), COS7 cells at 24 h posttransfection, or HDF cells at 8 × 10^5^ per 100 mm dish, were washed three times with phosphate-buffered saline (PBS) and scraped into 200-µL SDS-PAGE sample buffer containing 100 mM DTT (SB-DTT). Alternatively, cells were washed three times in PBS, placed on ice, and incubated with 500 µL of 2% sodium deoxycholate in PBS containing protease inhibitors (Complete Mini; Roche, Cat. No. 11836153001) for 10 min. The deoxycholate (DOC)-soluble lysate was collected and centrifuged at 10,000 r.c.f for 10 min to remove particulate material, mixed with SB-DTT, and stored at −20°C. The remaining DOC-insoluble material was removed with SB-DTT and scraping, and also stored. ECM produced by HDF was isolated from 3 × 100 mm dishes after 2 days culture as described previously (28) and extracted with 100 µL boiling-hot SB-DTT and scraping across the three plates. Conditioned media (CM) was collected from 5 × 100 mm plates after 48 h culture, centrifuged at 400 r.c.f for 5 min to remove cell debris, filtered through a 0.22 µm pore sterile filter unit (Millipore, Cat. No. SLGP033RB), transferred to a fresh tube, and incubated with 40 µL Heparin-agarose (Sigma, Cat. No. H6508) and protease inhibitors for 2 h at 4°C under rotation. Beads were washed three times in 10 mL Tris-buffered saline (TBS) containing 2 mM CaCl_2_. Bound proteins were eluted in SB-DTT. Samples were separated on 10% SDS-PAGE gels under reducing conditions, transferred to PVDF membrane (pore size 0.2 µm, Merck Millipore, Cat. No. ISEQ00010) and probed with antibodies as indicated in the text, with primary antibodies incubated overnight at 4°C and secondary antibodies for 1 h at room temperature, with rotation. The chemiluminescent signal was produced using ECL Western Blotting Detection Reagents (Amersham, Cat. No. RPN2209) and detected with either High Performance Chemiluminescence film (Amersham, Cat. No. 28906837) and a Curix 60 film processor (AGFA), or with a G:Box XRQ Western blot imager (Syngene) and GeneSys software (Syngene).

### Immunofluorescence Microscopy

COS7 or HDF cells cultured on coverslips for 2 or 48 h were washed three times in PBS and either fixed in 4% paraformaldehyde (PFA; Alfa Aesar, Cat. No. 43368) in PBS for 10 min (nonpermeabilized cells) or permeabilized in 0.5% Triton X-100 in PBS for 10 min and then fixed (permeabilized cells). Primary and secondary antibodies (Table 1) were applied in a humidified chamber for 1.5 and 1 h, respectively, with washing in PBS between each step. Coverslips were washed in water and mounted on slides in Vectashield with DAPI (Vector, Cat. No. H-1200). Cells were examined with a Leica SP5-AOBS confocal laser scanning microscope attached to a Leica DM16000 inverted epifluorescence microscope with HCX Plan Apo ×63 N.A.1.4 oil immersion objective lens, using Leica confocal software version 2.5. Images were acquired either as XY sections, or XYZ stacks, at *zoom 1* or *2*, at room temperature. For HyVolution2 imaging, a Leica SP8 AOBS confocal laser scanning microscope attached to a Leica DMi8 inverted epifluorescence microscope (Heidelberg, Germany) with a ×63 HC PL APO CS2 N.A. 1.4 oil immersion lens was used. Image stacks were acquired with pixel resolution optimized for superresolution imaging (36 × 36 × 130 nm) and deconvolution performed using Huygens software (Scientific Volume Imaging). Pearson’s correlation for colocalization was performed using Volocity software, Version 6.3 (PerkinElmer) and calculated per cell. Cell areas were calculated using FIJI image analysis software (ImageJ 1.50e). The number of focal adhesions per cell was calculated using FIJI by setting a background threshold for vinculin intensity and measuring the number of particles between 2 and 20 µm^2^ within each image, divided by the cell number as determined by counting nuclei.

### Biotin Ligase Proximity-Dependent Ligation and Proteomics

COS7 cells (8 × 10^5^ per 100 mm dish) were transfected with TSP1.BirA-HA plasmid as described above, then treated 2 h later with 50 µM biotin (Sigma, Cat. No. B4501) (24) for 16 h. Triton X-100 extracts were isolated by either trypsinizing the cells, washing three times in PBS, and lysing the cell pellet in 0.5% Triton X-100 for 10 min at 4°C (interactome 1) or washing the cells in PBS and incubating with 0.5% Triton X-100 for 10 min at 4°C (interactome 2). All Triton X-100-soluble cell extracts were separated by SDS-PAGE and the gel lanes subjected to in-gel tryptic digestion with a DigestPro automated digestion unit (Intavis Ltd.). The resulting peptides were fractionated in an Ultimate 3000 nano-LC system in line with an LTQ-Orbitrap Velos mass spectrometer (Thermo Scientific). This experimental design was repeated three times. In brief, peptides in 1% (vol/vol) formic acid were injected onto an Acclaim PepMap C18 nanotrap column (Thermo Scientific). After being washed with 0.5% (vol/vol) acetonitrile 0.1% (vol/vol) formic acid, peptides were resolved on a 250 mm × 75 μm Acclaim PepMap C18 reverse phase analytical column (Thermo Scientific) over a 150 min organic gradient, using seven gradient segments (1%–6% solvent B over 1 min, 6%–15% B over 58 min, 15%–32% B over 58 min, 32%–40% B over 5 min, 40%–90% B over 1 min, held at 90% B for 6 min and then reduced to 1% B over 1 min) with a flow rate of 300 nL/min. Solvent A was 0.1% formic acid and Solvent B was aqueous 80% acetonitrile in 0.1% formic acid. Peptides were ionized by nanoelectrospray ionization at 2.1 kV using a stainless-steel emitter with an internal diameter of 30 μm (Thermo Scientific) and a capillary temperature of 250°C. Tandem mass spectra were acquired using an LTQ- Orbitrap Velos mass spectrometer controlled by Xcalibur 2.0 software (Thermo Scientific) and operated in data-dependent acquisition mode. The Orbitrap was set to analyze the survey scans at 60,000 resolution (at *m*/*z* 400) in the mass range *m*/*z* 300–2,000 and the top 20 multiply charged ions in each duty cycle selected for MS/MS in the LTQ linear ion trap. Charge state filtering, where unassigned precursor ions were not selected for fragmentation, and dynamic exclusion (repeat count, 1; repeat duration, 30 s; exclusion list size, 500), were used. Fragmentation conditions in the LTQ were as follows: normalized collision energy, 40%; activation q, 0.25; activation time 10 ms; and minimum ion selection intensity, 500 counts. The raw data files were processed and quantified using Proteome Discoverer software v1.4 (Thermo Scientific) to run a Mascot search against the SwissProt Human database. Peptide precursor mass tolerance was set at 10 ppm, and MS/MS tolerance was set at 0.8 Da. Search criteria included carbamidomethylation of cysteine (+57.0214) as a fixed modification and oxidation of methionine (+15.9949) and biotinylation of lysine (+226.295 Da) as variable modifications. Searches were performed with full tryptic digestion and a maximum of two missed cleavages was allowed. The reverse database search option was enabled and all peptide data was filtered to satisfy false discovery rate (FDR) of 5%.

### In Situ Proximity Ligation

HDF or COS7 cells were prepared as described for indirect immunofluorescence up to incubation with primary antibody. Then the Duolink (Sigma) protocol was performed according to manufacturer’s instructions with Duolink In Situ PLA Probes anti-Mouse MINUS (Cat. No. DUO92004) and anti-Rabbit PLUS (Cat. No. DUO92002) secondary antibodies and green detection reagents (Cat. No. DUO92014). Cells were examined by widefield fluorescence microscopy under a Leica DMIRE2 inverted microscope, equipped with electronically controlled shutters and filter wheel. Images were captured under the ×63 oil immersion objective (NA 1.4) using a Hamamatsu camera controller C4742_95 run by Leica Applications Systems software (version 4.5.0). Analysis of in situ proximity ligation signal was performed with FIJI image analysis software by setting a threshold color brightness (as determined by background fluorescence) of 30, and measuring the total area of fluorescence above the threshold within an image divided by cell number (counted from DAPI-stained nuclei). At least 59 cells per experiment from each of three separate experiments were analyzed.

### In Vitro TSP1 Binding Assays

Recombinant human PDIA3/ERp57 or CCN2 proteins were coated at various concentrations onto wells of Immulon 2Hb plates (ThermoFisher, Cat. No. 3455) in TBS containing 2 mM CaCl_2_ and 15 µM ZnSO_4_ [incubation buffer (IB)], for 16 h at 4°C. In some experiments, pepsin-digested calf skin-derived collagen I (Sigma, Cat. No. C-3511) was coated as a positive control for TSP1 binding at a saturating concentration of 10 µg/mL (36). Other wells contained bovine serum albumin (BSA; Sigma, Cat. No. A9647) as a negative control. All following steps were at room temperature. Wells were washed in TBS containing 2 mM CaCl_2_, 15 µM ZnSO_4_, 0.1% BSA and 0.1% Tween20 [wash buffer (WB)] three times and blocked in WB containing 5% BSA for 1 h. After three washes in WB, 50 µg/mL (388 µM) of native human TSP1 in IB was added for 1 h. After three washes in WB, anti-TSP1 (C9) in IB was added at 1 µg/mL for 1 h. After three washes in WB, anti-mouse-IgG-horseradish peroxidase (HRP) in IB was added at 0.075 µg/mL for 1 h. After three washes in WB, wells were incubated for 15 min with 0.4 g/L Turbo 3,3′ 5,5′-tetramethylbenzidine (TMB) substrate and 0.02% hydrogen peroxide in citric acid buffer (Pierce, Cat. No. 34021). The colorimetric reaction was stopped by addition of 2 M H_2_SO_4_ and the absorbance in the wells measured at 450 nm in a plate reader (M2/spectra max; Molecular Devices). The experiment was repeated three times.

### Isolation of Sterile Conditioned Media and ECM for Cell Culture

Mouse embryonic fibroblasts (MEFs; WT or *Pdia3^−/−^*) (27) were plated at 2.5 × 10^5^ cells per 60 mm dish on coverslips for 48 h. Conditioned medium (CM) was then removed, centrifuged at 400 r.c.f for 5 min, filtered through a 0.22 µm pore sterile filter unit, and retained on ice. The ECM was isolated under sterile conditions as described previously (28). WT or *Pdia3^−/−^* MEFs cells were plated on the isolated ECM, or with CM, on coverslips at 5 × 10^5^ cells per 60 mm dish. After 2 h, cells were washed three times with PBS and prepared for immunofluorescence as described above. In experiments where MEFs were pretreated with 16F16, 2.5 × 10^5^ cells were plated per 60 mm dish and treated with 1 µM 16F16 at 2 h after plating. After 24 h, MEFs were replated on coverslips in a 60 mm dish and retreated with 1 µM 16F16 for 16 h. Then CM was prepared and ECM isolated as described above. In some experiments, CM was then incubated with 40 µL heparin-agarose (Sigma, Cat. No. H6508) beads, or agarose-only resin (Pierce, Cat. No. 26150) as a negative control, at 4°C with rotation for 1 h. Each CM was centrifuged at 1,000 r.c.f for 5 min and the supernatant filtered through a 0.22 µm pore sterile filter unit before adding with 5 × 10^5^ *Pdia3^−/−^* MEFs onto coverslips in 60 mm dishes for 2 h. In some experiments, recombinant CCN2 was added at a range of final concentrations to CM from *Pdia3^−/−^* MEFs and coplated with 5 × 10^5^ *Pdia3^−/−^* MEFs on coverslips in 60 mm dishes and incubated for 2 h.

### Analysis of the Heparin-Binding Secretome: TMT Proteomics

WT-MEFs and *Pdia3^−/−^* MEFs were plated at 8 × 10^5^ cells per 100-mm dish in five dishes and cultured for 48 h. CM was treated as described under *Cell Fractionation and Immunoblotting*, with 40-µL heparin-agarose. The heparin-agarose-bound samples were separated by SDS-PAGE and the gel lane subjected to in-gel tryptic digestion using a DigestPro automated digestion unit (Intavis Ltd.). The resulting peptides were labeled with Tandem Mass Tag (TMT) six plex reagents according to manufacturer’s protocol (Thermo Fisher Scientific) and the labeled samples pooled. This pooled sample was then desalted using a SepPak cartridge according to the manufacturer’s instructions (Waters). Eluate from the SepPak cartridge was evaporated to dryness, resuspended in 1% (vol/vol) formic acid, and fractionated using an Ultimate 3000 nano-LC system in line with an Orbitrap Fusion Tribrid mass spectrometer (Thermo Scientific). In brief, the peptides were injected onto an Acclaim PepMap C18 nanotrap column (Thermo Scientific). After being washed with 0.5% (vol/vol) acetonitrile 0.1% (vol/vol) formic acid, peptides were resolved on a 250 mm × 75 μm Acclaim PepMap C18 reverse phase analytical column (Thermo Scientific) over a 150 min organic gradient, using 6 gradient segments (5%–9% solvent B over 2 min, 9%–25% B over 94 min, 25%–60% B over 23 min, 60%–90% B over 5 min, held at 90% B for 5 min and then reduced to 1% B over 2 min) with a flow rate of 300 nL/min. Solvent A was 0.1% formic acid and Solvent B was aqueous 80% acetonitrile in 0.1% formic acid. Peptides were ionized by nanoelectrospray ionization at 2.0 kV using a stainless-steel emitter with an internal diameter of 30 μm (Thermo Scientific) and a capillary temperature of 275°C.

All spectra were acquired using an Orbitrap Fusion Tribrid mass spectrometer controlled by Xcalibur 2.0 software (Thermo Scientific) and operated in data-dependent acquisition mode using an SPS-MS3 workflow. FTMS1 spectra were collected at a resolution of 120,000, with an automatic gain control (AGC) target of 400,000 and a max injection time of 100 ms. Precursors were filtered with an intensity range from 5,000 to 1E20, according to charge state (to include *charge states 2–6*) and with monoisotopic precursor selection. Previously interrogated precursors were excluded using a dynamic window (60 s ±10 ppm). The MS2 precursors were isolated with a quadrupole mass filter set to a width of 1.2 *m*/*z*. ITMS2 spectra were collected with an AGC target of 10,000, max injection time of 70 ms and collision-induced dissociation (CID) collision energy of 35%.

For FTMS3 analysis, the Orbitrap was operated at 30,000 resolution with an AGC target of 50,000 and a max injection time of 105 ms. Precursors were fragmented by high energy collision dissociation (HCD) at a normalized collision energy of 55% to ensure maximal TMT reporter ion yield. Synchronous Precursor Selection (SPS) was enabled to include up to 5 MS2 fragment ions in the FTMS3 scan. The raw data files were processed and quantified using Proteome Discoverer software v2.1 (Thermo Scientific) and searched against the UniProt Mouse database using the SEQUEST algorithm. Peptide precursor mass tolerance was set at 10 ppm, and MS/MS tolerance was set at 0.6 Da. Search criteria included oxidation of methionine (+15.9949) as a variable modification and carbamidomethylation of cysteine (+57.0214) and the addition of the TMT mass tag (+229.163) to peptide N-termini and lysine as fixed modifications. Searches were performed with full tryptic digestion and a maximum of two missed cleavages were allowed. The reverse database search option was enabled and the data were filtered to satisfy false discovery rate (FDR) of 5%. Experiments were replicated three times. Proteins that were at least twofold changed across all three experiments were analyzed against the Molecular Signatures database with reference to the Gene Ontology, hallmarks and cancer modules gene sets at default parameters (29) (http://software.broadinstitute.org/gsea/msigdb/). The STRING functional protein association database (https://string-db.org/) (30) was searched with reference to interaction evidence from textmining, experiments, databases, and neighborhood with requirement for high-confidence interaction scores.

### Immunoprecipitation

WT-MEFs or *Pdia3^−/−^* MEFs were plated at 2.5 × 10^5^ cells per 60 mm dish (for CM) or 8 × 10^5^ cells per 100 mm dish (for 0.5% Triton X-100-soluble lysate) and cultured for 48 h. Recombinant protein G (rProteinG) agarose beads (Invitrogen, Cat. No. 15920-010; at 40 µL per sample) were washed three times in 10 mL TBS containing 2 mM CaCl_2_ and protease inhibitors before incubating with 2 µg anti-CCN2 or 1 µg antifibronectin antibody (Table 1) or nonimmune IgG control (mouse IgG, Santa Cruz, Cat. No. sc-2025, rabbit IgG, Sigma, Cat. No. I5006), at 4°C with rotation for 1 h. The beads were then pelleted and washed three times in 10 mL TBS containing 2 mM CaCl_2_ and protease inhibitors and retained on ice. CM was collected from one 60 mm dish and centrifuged at 400 r.c.f. for 5 min. Protease inhibitors were added to the supernatant before incubation with the antibody-coated rProtein G-agarose beads with rotation for 1 h at 4°C. For cell lysates, Triton X-100 extracts were prepared (as described in *Biotin Ligase Proximity-Dependent Ligation and Proteomics*), and the supernatant diluted to 10 mL and incubated with rProtein G-agarose beads bound to antibody with rotation for 1 h at 4°C. Then, beads were pelleted and washed three times in 10 mL TBS containing 2 mM CaCl_2_ and protease inhibitors before elution of bound proteins in 50-µL SDS-PAGE sample buffer and analysis by SDS-PAGE and immunoblotting.

### Statistical Analysis

Numbers of independent experiments are stated in each figure legend. Data are presented as bar charts or scatter dot plots with a horizontal line to show the mean, unless otherwise stated in the legend and were plotted with Prism software. All values from each replicate experiment are plotted unless stated otherwise. In data sets with many datapoints, the Shapiro-Wilks normality test was used to test for Gaussian distribution. The statistical tests used are stated in the figure legends and were based on whether two or more conditions were compared, whether data were normally distributed, and/or standard deviations were equivalent or not. Kruskal–Wallis outputs were analyzed by Dunn’s multiple comparison test. ANOVA outputs were analyzed by Dunnett’s multiple comparison test.

## RESULTS

### Identification of PDIA3 as an Intracellular Associate of TSP1

The method of biotin ligase proximity-dependent ligation (also known as BioID) utilizes a promiscuous biotin ligase, BirA, fused to a protein of interest, to achieve biotinylation of associated proteins within a radius of ∼9 nm (24). We used BioID to gain new insights into the intracellular interactions of TSP1.

**Figure 1.**
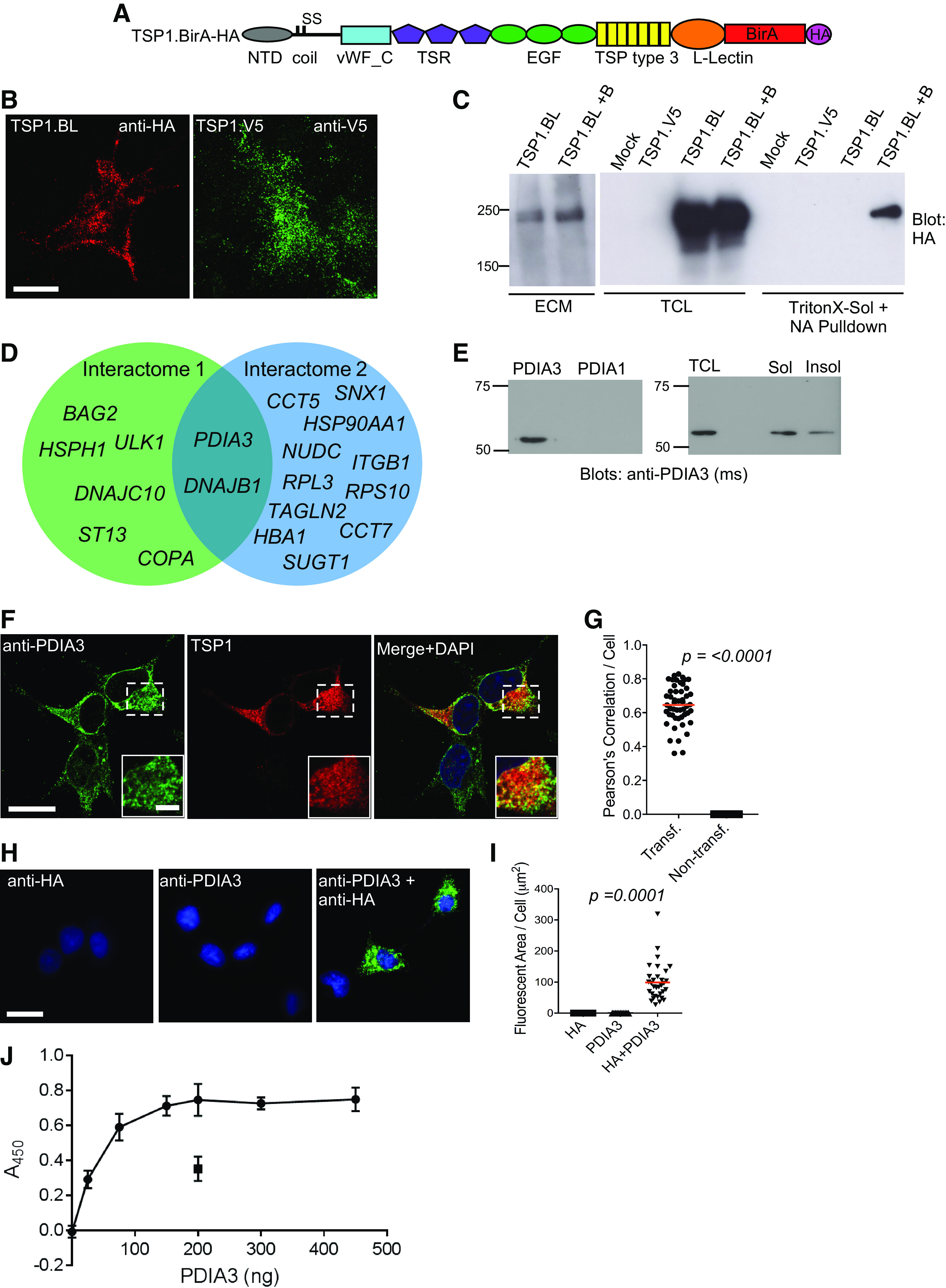
Identification and validation of PDIA3 as an interaction partner of TSP1. *A*: domain diagram of the TSP1-bacterial BirA biotin ligase fusion protein. *B*: TSP1.BirA-HA (TSP1-BL) and TSP1.V5 are both deposited into ECM with a punctate pattern, as visualized by indirect immunofluorescence of ECM isolated from transfected COS7 cells. Scale bar = 20 µm. *C*: detection of TSP1.BirA-HA (TSP1-BL) protein in ammonium hydroxide-extracted ECM of COS7 cells, in total cell lysate (TCL), and in neutravidin (NA) pulldown from Triton X-100-soluble cell extracts. Cells were incubated without or with (+B) biotin. Molecular mass markers are in kDa. *D*: Venn diagram of gene products identified by mass spectrometry from BirA proximity-dependent ligation. Interactomes 1 and 2 refer to two separate cell extraction protocols (see materials AND METHODS). *E*: *left*: specificity of a mouse (ms) PDIA3 antibody used in this study. 50 ng of recombinant protein was loaded in each lane. *Right*: detection of PDIA3 protein in TCL and Triton X-100-soluble (Sol) and -insoluble (Insol) extracts of COS7 cells. *F*: indirect immunofluorescent colocalization of PDIA3 and TSP1.BirA-HA in COS7 cells, imaged by confocal microscopy and presented as an XY slice. Scale bars = 20 µm (inset = 5 µm). *G*: quantitative analysis of PDIA3 and TSP1.BirA-HA colocalization in transfected COS7 cells, by Pearson’s correlation (*n* = 3 experiments). *H*: representative images of in situ proximity ligation of PDIA3 and TSP1.BirA-HA in transfected COS7 cells. Scale bar = 20 µm. *I*: quantitative analysis of fluorescent area per cell from images as in *H*; statistical analysis by Kruskal-Wallis test. The full set of controls for *H* and *I* are shown in Supplemental Fig. S1. *J*: in vitro binding of TSP1 to surface-bound PDIA3. 200 ng of collagen I (boxed) was used a positive control for TSP1 binding. Datapoints show means and error bars show standard deviation. All experiments *n* = 3. BirA, bifunctional ligase/repressor; coil, coiled-coil; ECM, extracellular matrix; EGF, epidermal growth factor-like domain; HA, hemagglutinin epitope; L-lectin, L-type lectin-like domain; NTD, NH_2_-terminal domain; SS, position of cysteines that form inter-subunit disulfide bonds; TSP type 3, thrombospondin type 3 repeats; TSR, thrombospondin type 1 domain; vWF_C, von Willebrand factor_type C domain.

An expression plasmid encoding a hemagglutinin-tagged, fusion protein, designated TSP1.BirA-HA (Fig. 1*A*), was engineered and tested for appropriate secretion and ECM deposition of TSP1.BirA-HA up on transient expression in COS7 cells. Typically, TSPs deposit into ECM in the form of puncta (31, 32) and, by immunofluorescence of isolated ECM, TSP1.BirA-HA also deposited in characteristic punctate arrays (Fig. 1*B*). The TSP1.BirA-HA protein was of the expected molecular weight in both total cell lysate (TCL) and isolated ECM and its abundance was not altered by addition of biotin to the culture media (Fig. 1*C*). When COS7 cells expressing TSP1.BirA-HA were incubated with 50 µM biotin for 16 h, TSP1-BirA-HA was efficiently isolated from Triton X-100-soluble cell extracts on neutravidin beads, demonstrating both effective BirA ligase activity and that TSP1-BirA-HA was self-biotinylated (Fig. 1*C*).

To identify TSP1-interacting proteins, Triton X-100-soluble extracts were prepared by two different methods (Interactomes 1 and 2; see materials AND METHODS) from COS7 cells expressing TSP1.BirA-HA and cultured either in the absence or presence of biotin for 16 h. Biotinylated proteins from each sample were enriched by neutravidin-affinity binding, resolved by SDS-PAGE, digested in-gel and analyzed by mass spectrometry. A total of 8 proteins were identified reproducibly in interactome 1s and 13 proteins in interactome 2, from 3 separate experiments for each method. The proteins from interactome 2 included a previously identified TSP-1-interacting protein, integrin β1 (33) (Fig. 1*D* and Table 2). Two proteins were identified by both experimental methods: protein disulfide isomerase A3 (PDIA3, also known as ERp57) and DNAJ homolog subfamily B member 1 [DNAJB1, also known as heat-shock protein 40 (HSP40); Fig. 1*D*]. PDIA3 is a member of the protein disulfide isomerase family that has functions in folding and disulfide bond formation of certain glycoproteins, including the ECM component fibronectin (34–36). As such, PDIA3 was prioritized for further analysis.

**Table 2.** Proteins identified from BioID proteomics analysis

			Expt 1			Expt 2			Expt 3		
	Protein	Gene	Score	Cov	Pep	Score	Cov	Pep	Score	Cov	Pep
Interactome 1	BAG family molecular chaperone regulator 2	*BAG2*	17.93	24.17	4	7.26	18.48	3	20.17	27.96	4
	Coatomer subunit α	*COPA*	14.05	5.15	6	1.76	0.90	1	9.88	4.98	5
	**DnaJ homolog subfamily B member 1**	***DNAJB1***	**8.46**	**12.94**	**4**	**12.33**	**10.88**	**3**	**6.76**	**5.31**	**1**
	DnaJ homolog subfamily C member 10	*DNAJC10*	5.19	10.41	4	9.53	6.31	4	10.95	9.58	7
	Heat shock 105 kDa/110 kDa protein 1	*HSPH1*	10.48	8.23	5	11.24	4.42	3	21.21	12.29	7
	Hsc70-interacting protein	*ST13*	13.39	25.34	3	0.00	4.79	1	13.60	27.40	3
	**Protein disulfide-isomerase A3**	***PDIA3***	**6.46**	**8.96**	**4**	**22.34**	**17.29**	**6**	**5.81**	**4.79**	**2**
	Serine/threonine-protein kinase ULK1	*ULK1*	1.97	0.67	1	7.68	0.67	1	3.87	0.67	1
Interactome 2	40S ribosomal protein S10	*RPS10*	51.73	5.45	1	43.93	5.45	1	49.81	5.45	1
	60S ribosomal protein L3	*RPL3*	35.98	6.84	1	57.51	1.74	1	28.28	16.52	1
	**DnaJ homolog subfamily B member 1**	***DNAJB1***	**109.8**	**10.59**	**4**	**112.7**	**14.12**	**4**	**20.28**	**2.06**	**1**
	Heat shock protein HSP 90-α	*HSP90AA1*	241.7	17.21	5	302.7	13.8	5	182.9	12.16	2
	Hemoglobin subunit α	*HBA1*	38.16	10.56	1	34.68	10.56	1	32.7	10.56	1
	Integrin β1	*ITGB1*	48.31	1.25	1	46.68	1.25	1	60.59	5.14	3
	Nuclear migration protein nudC	*NUDC*	154.8	21.75	6	133.3	22.66	7	99.66	16.31	5
	**Protein disulfide-isomerase A3**	***PDIA3***	**62.53**	**4.16**	**2**	**60.98**	**6.53**	**3**	**51.57**	**8.71**	**4**
	Sorting nexin-1	*SNX1*	103.6	10.34	5	93.28	7.47	4	50.46	1.92	1
	Suppressor of G2 allele of SKP1 homolog	*SUGT1*	119.1	15.62	4	107	15.89	4	46.25	8.22	2
	T-complex protein 1 subunit epsilon	*CCT5*	44.94	6.47	2	74.54	7.21	4	107.3	10.54	5
	T-complex protein 1 subunit eta	*CCT7*	102	5.52	3	60.97	4.42	2	120.1	15.65	7
	Transgelin-2	*TAGLN2*	67.72	11.56	2	65.84	23.62	4	115.6	16.58	3

Score is sum of the ion scores for each unique peptide using Mascot software. Proteins identified by both experimental designs are bolded. Cov, percentage of the protein sequence covered by identified peptides; Pep, unique peptides.

We have validated certain commercial antibodies for specificity against recombinant PDIA3 protein versus recombinant PDI/PDIA1 (37). An additional anti-PDIA3 raised in mouse was validated here (Fig. 1*E*). The presence of PDIA3 in nontransfected COS7 cells was confirmed by immunoblotting with this antibody (Fig. 1*E*).

### Validation of PDIA3 as a Novel Intracellular Interaction Partner of TSP1

PDIA3 is mostly located in the endoplasmic reticulum (ER) (38) but has also been reported as an extracellular protein (34, 39). To examine the localization of PDIA3 in COS7 cells and validate the association of PDIA3 and TSP1, localization of TSP1.BirA-HA and endogenous PDIA3 was investigated by confocal microscopy (Fig. 1*F*). PDIA3 and TSP1.BirA-HA colocalized in the perinuclear region with a mean Pearson’s correlation of ∼0.65 (Fig. 1*G*) and extracellular PDIA3 was not apparent by this method. Association of TSP1 and PDIA3 in COS7 cells was also examined by in situ proximity ligation, which yields a fluorescent signal when two target proteins are within 40 nm (40). The signal (quantified as fluorescent area/cell) from COS7 cells expressing TSP1.BirA-HA and stained with antibodies to both PDIA3 and HA was significantly increased over the signals obtained with separate single antibody stainings (Fig. 1, *H* and *I*), or on costaining of PDIA3 and a control protein, vimentin (Supplemental Fig. S1; all Supplemental Figures are available at https://doi.org/10.6084/m9.figshare.14852661). Thus, PDIA3 and TSP1.BirA-HA are in close proximity within COS7 cells.

In view that PDIA3 functions in the ER in a complex with calnexin and calreticulin (41), we tested whether TSP1 and PDIA3 interact directly by examining in vitro binding with purified proteins. Collagen I (boxed) was included as a positive control for TSP1 binding (42). TSP1 in solution bound in a saturable manner to surface-bound PDIA3 coated at different concentrations (Fig. 1*J*). Thus, PDIA3 and TSP1 can bind directly in the absence of other proteins.

### Endogenous TSP1 and PDIA3 Associate in the Endoplasmic Reticulum of Human Dermal Fibroblasts

**Figure 2.**
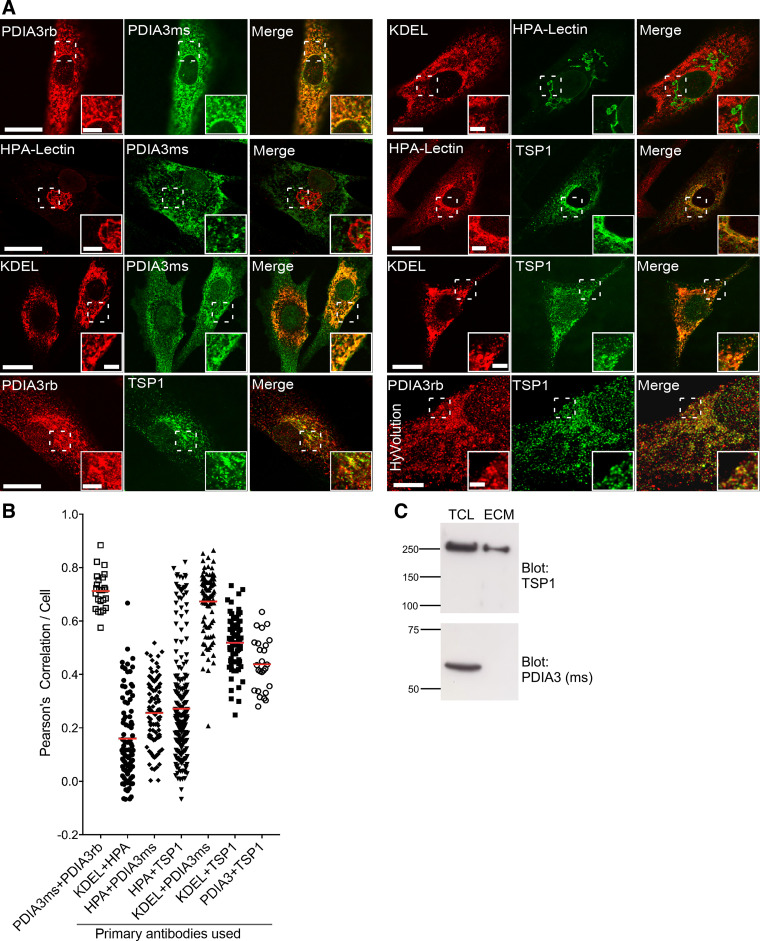
Colocalization of PDIA3 and TSP1 in the endoplasmic reticulum and vesicles of human dermal fibroblasts. *A*: representative images of the intracellular localizations of PDIA3 and TSP1 in human dermal fibroblasts (HDF). Anti-KDEL was included as a marker for ER and HPA-Lectin as a marker for Golgi apparatus. The bottom right-hand row of images was taken using HyVolution software; all others are XYZ stacks of confocal microscopy images. In confocal images, scale bar = 20 µm and inset scale = 5 µm. For HyVolution images, scale = 5 µm and inset scale = 1.25 µm. *B*: quantitative analysis of pairwise colocalizations, as indicated, in HDF cells, by Pearson’s correlation (*n* = 3 experiments). *C*: immunoblot analysis of the distribution of TSP1 and PDIA3 between TCL and isolated ECM of HDF cells. Markers are in kDa. In all panels, ms = mouse IgG and rb = rabbit IgG. ER, endoplasmic reticulum; HPA, *Helix pomatia* agglutinin; TCL, total cell lysates.

Because an increased abundance of PDIA3 has been associated with myofibroblast differentiation and fibrosis (34) and TSP1 is also associated functionally with fibrosis (12, 42), primary human dermal fibroblasts (HDF) were selected for experiments to examine the association of native TSP1 and PDIA3. First we validated the various reagents. PDIA3 antibodies raised in rabbit or mouse yielded highly correlated intracellular staining patterns with mean Pearson’s correlation of ∼0.7 (Fig. 2, *A* and *B*). The localizations of PDIA3 and intracellular TSP1 were then mapped with reference to specific markers for the Golgi apparatus (HPA-Lectin) or ER (KDEL antibody). As expected, HPA-Lectin and KDEL antibody had largely distinct (mean Pearson’s correlation of 0.16) staining patterns in HDF (Fig. 2, *A* and *B*). Costaining of anti-KDEL with either anti-TSP1 (mean Pearson’s of 0.52) or anti-PDIA3 (mean Pearson’s of 0.67) indicated localization of both proteins in the ER and also that neither TSP1 nor PDIA3 was entirely restricted to the ER (Fig. 2, *A* and *B*). However, as expected from its reported distribution as an ER protein, PDIA3 colocalized only weakly with HPA-Lectin (mean Pearson’s of 0.29). The production of TSP1 is related to active cell proliferation (43) and variable staining patterns were observed for TSP1: some cells had intense intracellular TSP1 staining whereas others had vesicular staining of lower intensity, and a wide range of correlation scores for TSP1 with HPA-Lectin were obtained (Fig. 2, *A* and *B*, mean Pearson’s of 0.25). Overall, cells with high intracellular TSP1 showed clear colocalization of anti-TSP1 with HPA-Lectin (Pearson’s ∼0.6–0.8), as expected for a protein destined for secretion.

Based on these control studies, costaining for TSP1 and PDIA3 was carried out (Fig. 2*A*) and revealed partial colocalization (Pearson’s correlation of ∼0.4; Fig. 2*B*), apparently in the ER and a subset of intracellular vesicles. Further investigation by superresolution confocal microscopy with HyVolution software clarified that PDIA3 and TSP1 colocalized mostly in ER-like structures adjacent to the nucleus and also in a minority of vesicles within the perinuclear region (Fig. 2*A*, *bottom right*).

Immunoblotting of TCL and ECM of HDF showed that PDIA3 was detected only in TCL, whereas, as expected, TSP1 was present both intracellularly and extracellularly (Fig. 2*C*). Overall, these results demonstrate that TSP1 and PDIA3 colocalize in HDF, predominantly within the ER.

### Functional Perturbation of PDIA3 Alters Secretion and ECM Deposition of Fibronectin and Thrombospondin-1 and Impacts Fibroblast Adhesion

**Figure 3.**
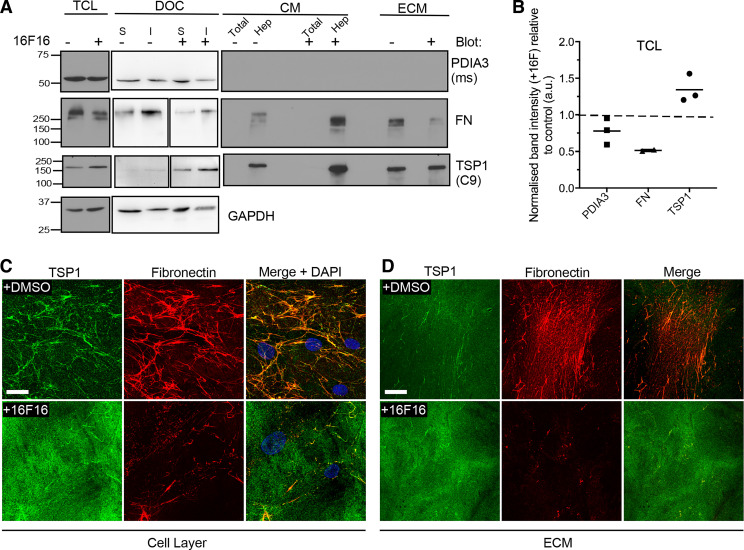
PDIA3 modulates secretion and ECM deposition of TSP1 and fibronectin by human dermal fibroblasts. *A*: HDF were treated with 1 µM 16F16 or DMSO as solvent control for 48 h. Immunoblots show the effect of PDIA3 inhibition on PDIA3, fibronectin (FN), and TSP1 protein abundance in total cell lysates (TCL); in 2% deoxycholate cell lysates (DOC), examined as DOC-soluble (S) and DOC-insoluble (I) fractions; conditioned media (CM), examined either as total CM or the heparin-affinity fraction (Hep), or isolated ECM. GAPDH was included as a loading control. *B*: quantified analysis of FN, PDIA3, and TSP1 proteins in TCL, normalized against GAPDH from multiple independent immunoblots of TCL samples. For each blot and primary antibody, the normalized control band intensity was set as 1 (indicated by dotted line on the graph) and the normalized band intensity of the +16F16 condition ratioed to the control. Horizontal bars show the mean, and each datapoint is from one experiment (*n* = 3 experiments in total). *C* and *D*: indirect immunofluorescence for TSP1 and fibronectin in nonpermeabilized cells (*C*), or isolated ECM (*D*), from HDF treated without or with 16F16, imaged by confocal microscopy and presented as XYZ stacks. *C* and *D* representative of 3 experiments. Scale bar = 20 µm. ECM, extracellular matrix.

To investigate whether the abundance or localization of TSP1 depends on PDIA3 function in HDF, we first used the compound 16F16 that inhibits PDIA3 (26). No fully selective inhibitor is available at this time. At 1 µM, 16F16 was not toxic to cells (Supplemental Fig. S2). Effects of PDIA3 inhibition on the abundance of PDIA3 or TSP1 were examined by immunoblotting, and fibronectin was also included as a control ECM protein identified previously to be dependent on PDIA3 (34). 16F16 treatment had no definite effect on the amount of PDIA3 in the cell fractions and PDIA3 was not detected in the CM or ECM (Fig. 3*A*, and quantified for TCL in Fig. 3*B*). Fibronectin was decreased in TCL after 16F16 treatment, and also in the deoxycholate (DOC)-soluble and -insoluble fractions, and in isolated ECM, yet was increased in CM (detected by enrichment through heparin-affinity pulldown; Fig. 3*A*, and quantified for TCL in Fig. 3*B*). In contrast, the amount of TSP1 was increased in 16F16-treated TCL, in both DOC fractions and in heparin-affinity pulldown of CM, although no difference was apparent in isolated ECM (Fig. 3*A* and quantified for TCL in Fig. 3*B*). These effects of 16F16 were examined further by indirect immunofluorescence of nonpermeabilized HDF (Fig. 3*C*). Around control HDF, TSP1 colocalized with discrete areas of fibronectin fibrils. 16F16-treated HDF had shorter and sparser networks of fibronectin fibrils than control cells and a diffuse, amorphous localization of TSP1 that mostly did not colocalize with residual fibronectin (Fig. 3*C*). Equivalent changes were apparent in isolated ECM: after 16F16 treatment, very little fibronectin was detected and TSP1 patterning was altered from punctate and fibrillar to diffuse, with little colocalization with fibronectin (Fig. 3*D*). Furthermore, immunoreactivity of TSP1 with the conformation-sensitive antibody D4.6 that binds to denatured, calcium-depleted TSP1 (44) was increased (data not shown). Thus, inhibition of PDIA3 leads to less fibronectin within the ECM and increased amounts of intracellular and secreted TSP1, yet the secreted TSP1 has an altered conformation and reduced colocalization with residual fibronectin in the ECM. The results from ECM also imply fibronectin-independent mechanisms for association of TSP1 into ECM.

**Figure 4.**
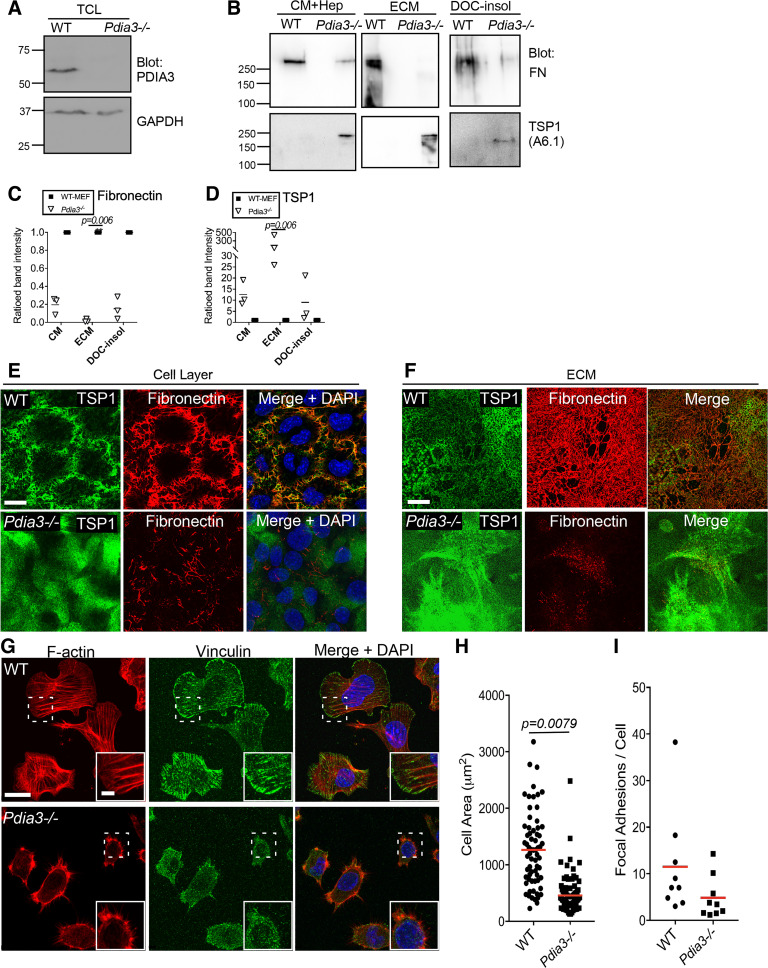
PDIA3 modulates ECM abundance and cell adhesion of mouse embryonic fibroblasts. *A*: immunoblot detection of PDIA3 protein in TCL of WT-MEFs but not *Pdia3^−/−^* MEFs. Blot reprobed with GAPDH as a loading control. *B*: immunoblots for fibronectin and TSP1 in CM, ECM, and 2% deoxycholate-insoluble (DOC-insol) fractions from WT-MEFs and *Pdia3^−/−^* MEFs. Molecular mass markers are in kDa. *C* and *D*: quantified analysis of protein levels from multiple immunoblots of the WT and *Pdia3^−/−^* MEFs cell fractions, probed for fibronectin (*C*) or TSP1 (*D*). Horizontal bars show the means, and each datapoint is from one experiment (*n* = 3 experiments in total). Statistical analysis by Kruskal-Wallis test. *E* and *F*: indirect immunofluorescence images of localizations of TSP1 and fibronectin around nonpermeabilized WT-MEFs or *Pdia3^−/−^* MEFs (*E*), or in isolated ECM of these cells (*F*), by confocal microscopy and presented as XYZ stacks. Scale bar = 20 µm. Representative of 3 experiments. *G*: confocal indirect immunofluorescence images of F-actin and vinculin in WT-MEFs and *Pdia3^−/−^* MEFs, 2 h after plating in serum-free media, presented as XYZ stacks. Scale bar = 20 µm and inset scale = 5 µm. Representative of 5 experiments. *H*: quantitative analysis of cell areas from immunofluorescence images of WT-MEFs and *Pdia3^−/−^* MEFs (*n* = 5 experiments). *I*: quantified analysis of focal adhesions per cell from indirect immunofluorescence for vinculin in WT-MEFs and *Pdia3^−/−^* MEFs (*n* = 5 experiments). Red bars show the mean; analysis of significance by Mann-Whitney test. CM, conditioned medium; ECM, extracellular matrix; MEFs, mouse embryonic fibroblasts; TCL, total cell lysate; WT, wild type.

Because 16F16 is not fully specific for PDIA3 (26) and to examine the effect of loss of PDIA3 activity by an independent approach, TSP1 and FN were compared in wild-type mouse embryonic fibroblasts (WT-MEFs) and strain-matched *Pdia*3*^−/−^* MEFs (27). The absence of PDIA3 protein in *Pdia3^−/−^* MEFs was confirmed by immunoblotting (Fig. 4*A*). Comparison of the abundance of TSP1 and FN in CM, isolated ECM, or in the deoxycholate-insoluble fraction [an extraction sample used to assess fibronectin within ECM (45)] of WT-MEFs or *Pdia3^−/−^* MEFs, identified that fibronectin was decreased in all the samples from *Pdia3^−/−^* MEFs (Fig. 4*B*, and quantified in Fig. 4*C*), whereas TSP1 was increased in CM and ECM from *Pdia3^−/−^* MEFs relative to WT-MEFs and was variably increased in the DOC-insoluble fraction from *Pdia3^−/−^* MEFs (Fig. 4*B*, and quantified in Fig. 4*D*). WT-MEFs established extensive fibrillar networks of fibronectin, whereas *Pdia3^−/−^* MEFs had reduced arrays of short, disorganized fibrils (Fig. 4*E*). TSP1 produced by WT-MEFs colocalized in part with fibronectin fibrils, whereas *Pdia3^−/−^* MEFs deposited widespread TSP1 puncta and patches that did not colocalize clearly with the residual fibronectin fibrils (Fig. 4*E*). These changes to fibronectin and TSP1 patterning were also apparent in isolated ECM from *Pdia3^−/−^* MEFs in comparison to ECM from WT-MEFs (Fig. 4*F*). The D4.6 antibody could not be used because it does not detect mouse TSP1.

These two lines of evidence (pharmacological inhibition and use of *Pdia3^−/−^* MEFs) both indicated that loss of PDIA3 function resulted in a distinct ECM microenvironment, depleted for fibronectin. In view that TSP1 and fibronectin have distinct effects on actin cytoskeleton in cell-substratum adhesion (46) and evidence that PDIA3-silencing or inhibition has effects on cell morphology (34, 37), the morphology of WT-MEFs and *Pdia3^−/−^* MEFs was examined and quantified under serum-free conditions with matched cell density and adhesion time (Fig. 4*G*). Whereas WT-MEFs spread and organized actin microfilaments and focal adhesions rapidly, over the same period *Pdia3^−/−^* MEFs reached significantly smaller cell areas (Fig. 4*G* and quantified in Fig. 4*H*), had fewer microfilament bundles in cell bodies and decreased numbers of focal adhesions (Fig. 4*G*; focal adhesions/cell are quantified in Fig. 4*I*). Thus, PDIA3 activity has a role in supporting initial fibroblast spreading and assembly of ECM-adhesions.

### Impaired Cell Adhesion of *Pdia3^−/−^* MEFs Depends on the Extracellular Microenvironment

**Figure 5.**
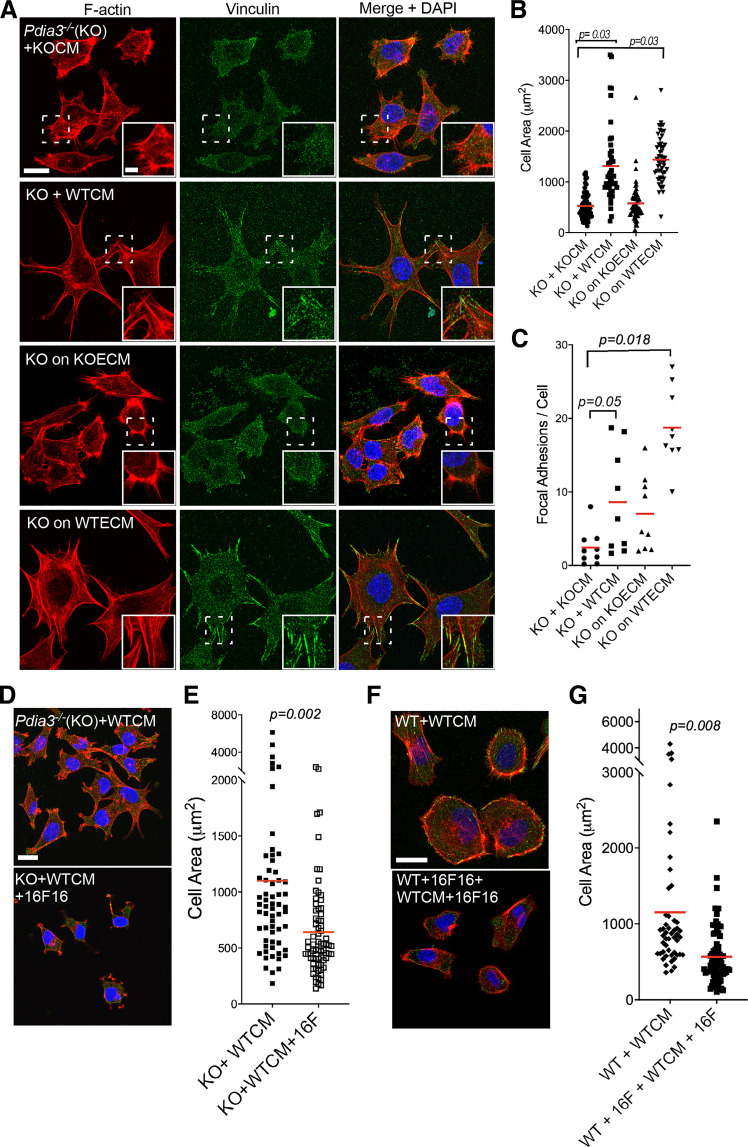
Conditioned media or extracellular matrix from WT-MEFs promotes adhesion of *Pdia3^−/−^* MEFs. *A*: representative immunofluorescence confocal images for F-actin and vinculin in *Pdia3^−/−^* MEFs (KO) fixed after 2 h adhesion in presence of either CM or isolated ECM from 48 h cultures of WT-MEFs (labeled as +WTCM or on WTECM, respectively) or equivalent samples from *Pdia3^−/−^* MEFs (labeled as KO + KOCM or KO on KOECM, respectively), presented as XYZ stacks. Scale bar = 20 µm (inset scale = 5 µm). *B*: quantified analysis of cell area from immunofluorescence images of *Pdia3^−/−^* MEFs under the same conditions as in *A* (*n* = 4 experiments). *C*: quantified analysis of focal adhesions per cell from immunofluorescence images for vinculin in *Pdia3^−/−^* MEFs under conditions as in *A* (*n* = 4 experiments). *B* and *C*: statistical analysis by Kruskal-Wallis test. *D* and *E*: inhibition of PDIA3 in WT-MEFs reduces the adhesion-promoting activity of WTCM on *Pdia3^−/−^* MEFs. *D*: representative confocal immunofluorescence images for F-actin and vinculin in *Pdia3^−/−^* MEFs after 2 h adhesion with CM from WT-MEFs grown for 48 h in the presence (+16 F) or absence of 1 µM 16F16, presented as XYZ stacks. Scale bar = 20 µm. *E*: quantified analysis of cell areas of *Pdia3^−/−^* MEFs from immunofluorescence images as in *D* and *n* = 3 experiments. *F* and *G*: inhibition of PDIA3 in WT-MEFs reduces the adhesion of WT-MEFs. *F*: representative confocal immunofluorescence images for F-actin and vinculin in WT-MEFs plated in the presence (WT + 16 F) or absence of 1 µM 16F16 for 2 h with CM from WT-MEFs grown for 48 h either in the presence (WTCM +16F16) or absence (WTCM) of 1 µM 16F16, presented as XYZ stacks. Scale bar = 20 µm. *G*: quantified analysis of cell area from immunofluorescence images as in *F* and *n* = 3 experiments. *E* and *G*: statistical analysis by unpaired *t* test with Welch’s test. In all graphs, red horizontal bars show the mean. CM, conditioned medium; ECM, extracellular matrix; KO, knockout; MEFs, mouse embryonic fibroblasts; WT, wild type; WTCM, conditioned medium from wild-type mouse embryonic fibroblasts; WTECM, extracellular matrix from wild-type mouse embryonic fibroblasts.

The alterations in relative abundance of extracellular TSP1 and fibronectin on loss of PDIA3 function and the reduced spreading and adhesion of *Pdia3^−/−^* MEFs raised the question whether PDIA3-dependent proteins of the secretome (i.e., proteins for which effective trafficking and secretion depends on PDIA3) could be responsible for the altered phenotype of *Pdia3^−/−^* MEFs. To investigate this, *Pdia3^−/−^* MEFs were plated for 2 h with CM or isolated ECM, each prepared from 48 h cultures of either WT-MEFs or *Pdia3^−/−^* MEFs. Cytoskeletal organization and cell spreading was examined after 2 h by immunofluorescence for F-actin and vinculin (Fig. 5*A*). *Pdia3^−/−^* MEFs (referred to as KO in figure labels) plated with either CM or ECM from WT-MEFs (referred to in figure labels as WTCM or WTECM, respectively) had larger areas (Fig. 5*B*), enhanced assembly of microfilament bundles, and more focal adhesions per cell than *Pdia3^−/−^* MEFs plated with either CM or ECM from *Pdia3^−/−^* MEFs (referred to in figure labels as KOCM or KOECM, respectively; Fig. 5, *A* and *C*). In contrast, WT-MEFs plated with either CM or ECM from *Pdia3^−/−^* MEFs had comparable spreading and F-actin organization to WT-MEFs plated with either WTCM or WTECM (Supplemental Fig. S3). Thus, the CM of *Pdia3^−/−^* MEFs is not enriched for active inhibitors of cell adhesion and spreading, whereas the CM of WT-MEFs contains factors that promote spreading and adhesion of *Pdia3^−/−^* MEFs.

To establish that the functional effects of WTCM on *Pdia3^−/−^* MEFs depended on PDIA3 activity, *Pdia3^−/−^* MEFs were plated with CM from WT-MEFs that had been preincubated with inhibitor 16F16. After 2 h of attachment, these *Pdia3^−/−^* MEFs had significantly smaller cell areas and few microfilament bundles compared with *Pdia3^−/−^* MEFs plated with control WTCM: indeed, the cell areas were similar to those of *Pdia3^−/−^* MEFs plated in CM from *Pdia3^−/−^* MEFs (Fig. 5, *D* and *E*, compared with Fig. 5*B*). The requirement for PDIA3 activity was examined further by testing WT-MEFs pretreated with 16F16 and then plated for 2 h in 16F16-treated WTCM. These cells also had smaller cell areas and fewer microfilament bundles compared with control WT-MEFs plated in WTCM (Fig. 5, *F* and *G*). From these results, we deduced that CM from WT-MEFs contains an activity to promote cell spreading and adhesion that is not present in CM from *Pdia3^−/−^* MEFs, and that this activity depends on the secreted products of fibroblasts with enzymatically-active PDIA3.

### Heparin-Binding Components of the PDIA3-Dependent Secretome Are Required for Cell Adhesion and Spreading

**Figure 6.**
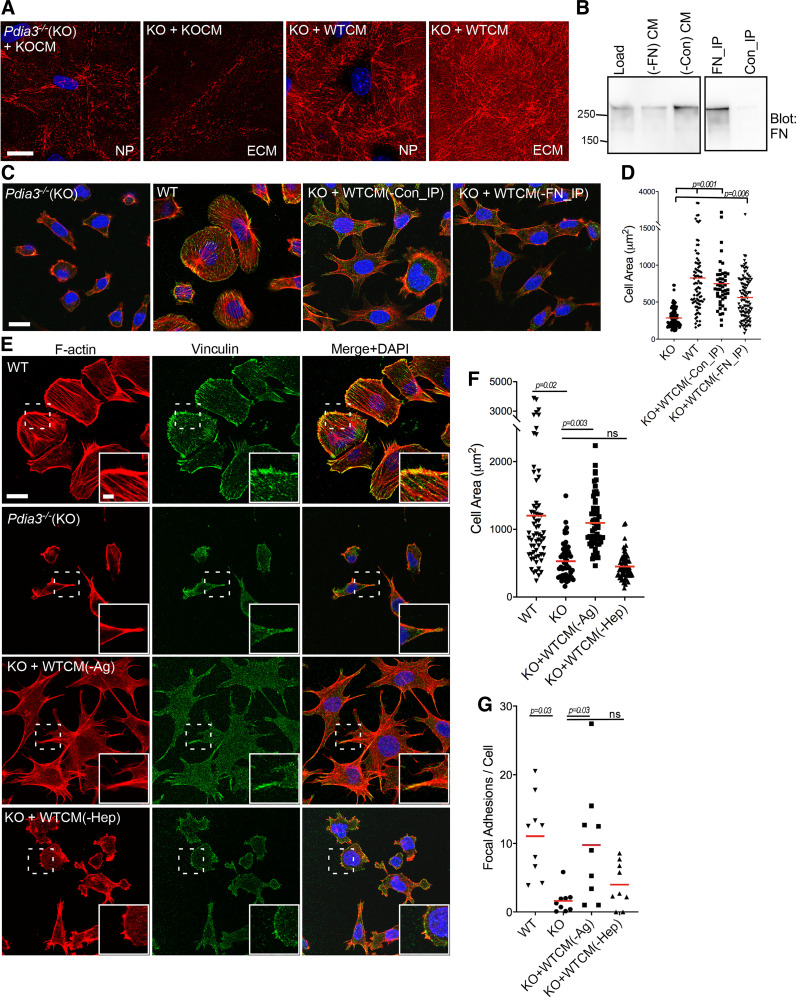
Identification that heparin-binding proteins in WTCM promote adhesion of *Pdia3^−/−^* MEFs. *A*: confocal immunofluorescence images of fibronectin around nonpermeabilized cells (NP), or in isolated ECM, for *Pdia3^−/−^* MEFs (KO) cultured for 48 h with CM from either *Pdia3^−/−^* MEFs (KO + KOCM) or from WT-MEFs (KO + WTCM), presented as XYZ stacks. Scale bar = 20 µm. Representative of 3 experiments. *B*: immunoprecipitation (IP) of fibronectin (FN) from WTCM from 48 h WT-MEFs cultures. Nonimmune rabbit IgG was used as a control (Con). Representative of 3 experiments. *C* and *D*: immunodepletion of fibronectin from WTCM has a negligible effect on the adhesion-promoting activity of WTCM for *Pdia3^−/−^* MEFs. *C*: confocal immunofluorescence images of *Pdia3^−/−^* MEFs (KO) or WT-MEFs (WT) stained for F-actin and vinculin (the merged images also include DAPI stain) after 2 h adhesion in serum-free media (shown in the two left panels) or for KO cells in WTCM immunoprecipitated as indicated (two right panels). Scale bar = 20 µm. *D*: quantified analysis of cell areas of WT-MEFs and *Pdia3^−/−^* MEFs, from images as in *C* and *n* = 3 experiments. *E*–*G*: depletion of heparin-binding proteins from WTCM reduces its activity to promote adhesion of *Pdia3^−/−^* MEFs. *E*: confocal immunofluorescence images of F-actin and vinculin (the merged images also include DAPI stain) in WT-MEFs (WT) or *Pdia3^−/−^* MEFs (KO) plated for 2 h in serum-free media (shown in the two top rows) or KO cells in the presence of control 48 h WTCM [WTCM(-Ag)], or WTCM depleted of heparin-binding proteins [WTCM(-Hep)], presented as XYZ stacks. Scale bar = 20 µm and in inset = 5 µm. *F*: quantified analysis of cell areas of WT-MEFs or *Pdia3^−/−^* MEFs (KO) under experimental conditions as in *E* from *n* = 3 experiments. *G*: quantified analysis of focal adhesions per cell from immunofluorescence images for vinculin as in *E* from *n* = 3 experiments. *D*, *F*, and *G*: red horizontal bars show the mean; statistical analysis by ANOVA test with Welch’s test. CM, conditioned medium; ECM, extracellular matrix; KO, knockout; MEFs, mouse embryonic fibroblasts; WT, wild type; WTCM, conditioned medium from wild-type mouse embryonic fibroblasts.

In view that either pharmacological inhibition or gene knockout of *Pdia3* reduced fibronectin abundance and that fibronectin is a major integrin ligand that promotes focal adhesions (47, 48), we first considered whether fibronectin is responsible for the adhesion-promoting activity of the CM from WT-MEFs on *Pdia3^−/−^* MEFs. *Pdia3^−/−^* MEFs plated for 2 h with WTCM had more cell-associated fibronectin fibrils than *Pdia3^−/−^* MEFs in CM from *Pdia3^−/−^* MEFs [Fig. 6*A*, nonpermeabilized (NP) samples], and this increase in fibronectin fibrils was also apparent in the isolated ECM (Fig. 6*A*, ECM samples). However, immunodepletion of fibronectin from WTCM (a representative example of CM immunodepletion is shown in Fig. 6*B*) led to only a minor alteration in the activity of WTCM to stimulate actin microfilament organization and spreading of *Pdia3^−/−^* MEFs (Fig. 6*C*, and quantified in Fig. 6*D*). Therefore, a separate approach was taken. Because many secreted, ECM-associated proteins, including TSP1 and fibronectin, contain heparin-binding domains (49–51), we investigated if heparin-binding extracellular proteins are required for the activity of WTCM. *Pdia3^−/−^* MEFs were plated for 2 h with WTCM that had been preincubated with either control agarose beads or heparin-agarose beads, and cell spreading and focal adhesions examined (Fig. 6*E*). Whereas WTCM preincubated with agarose-beads [identified as WTCM(-Ag) in Fig. 6, *E*–*G*] had activity equivalent to untreated WTCM (Fig. 5, *F* and *G*, compared with Fig. 5*B*), WTCM preincubated with heparin-agarose beads [identified as WTCM(-Hep) in Fig. 6, *E*–*G*] was clearly inactive for promotion of cell-body microfilament bundles and cell spreading (Fig. 6*E*; cell areas quantified in Fig. 6*F*), or focal adhesions (Fig. 6*E*, and quantified in Fig. 6*G*) in *Pdia3^−/−^* MEFs. Thus, heparin-binding proteins are crucial for the cell-spreading activity of WTCM on *Pdia3^−/−^* MEFs.

### Comparative Analysis of Heparin-Binding Secreted Proteins of WT-MEFs and *Pdia3^−/−^* MEFs

**Figure 7.**
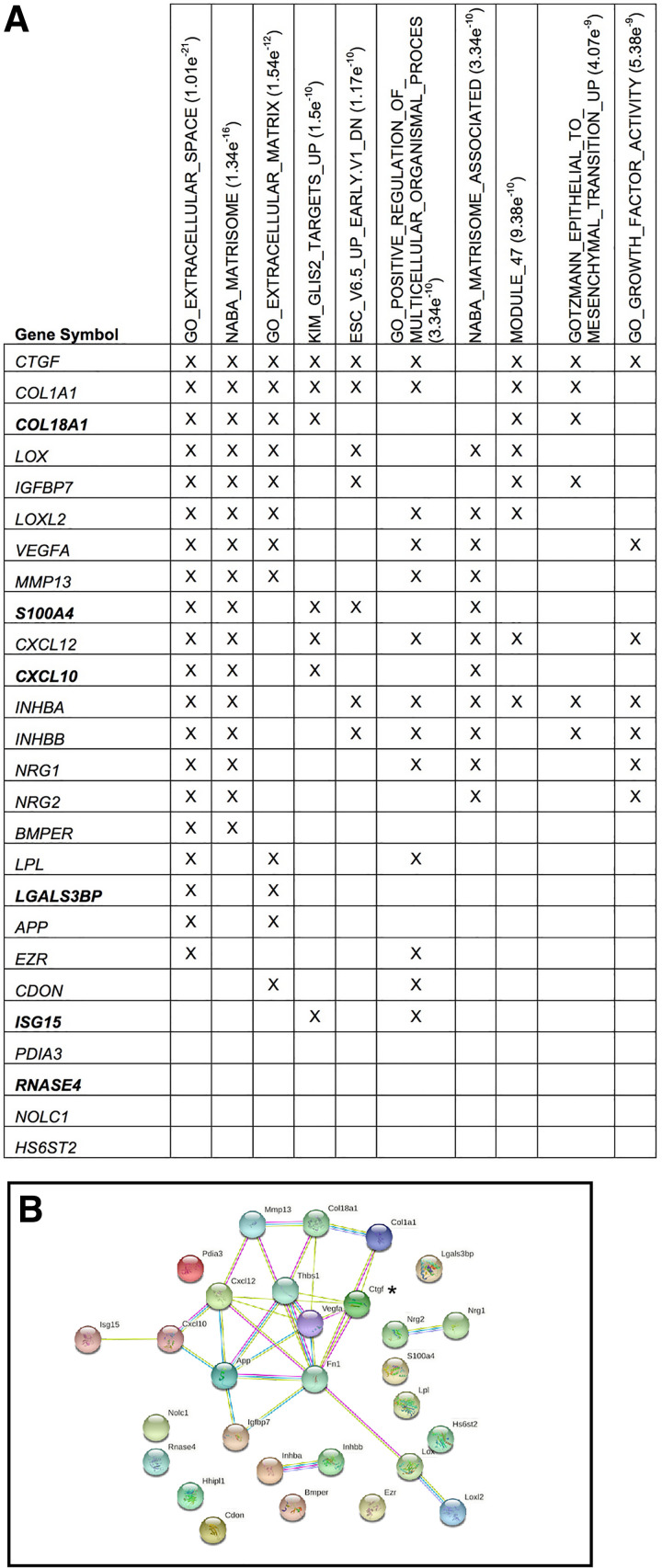
Correlations of identified PDIA3-dependent, heparin-binding, secreted proteins with ECM-related signatures and protein networks. *A*: top ten significant overlaps of gene signature sets of the Molecular Signatures database for the proteins at least twofold decreased (gene names in plain text) or increased (gene names in bolded text) in CM of *Pdia3^−/−^* MEFs relative to the CM of WT-MEFs, as identified by TMT proteomics. Details of the TMT proteomics data set are in Supplemental Table S1. *Hhlp1* is not included in the figure because it is not mapped in the Molecular Signatures database. *B*: STRING functional protein interaction network based on the above proteins plus TSP1 and fibronectin. Asterisk marks CCN2 (identified by the alternative gene name ctgf in the network output). CCN2, cell communication network 2; CM, conditioned medium; ECM, extracellular matrix; MEFs, mouse embryonic fibroblasts; TMT, tandem mass tag; WT, wild type.

To identify candidate adhesion-promoting proteins in WTCM, large-scale samples of the heparin-binding fractions of the secretomes of WT-MEFs or *Pdia3^−/−^* MEFs were prepared and compared quantitatively by tandem-mass-tagging and Orbitrap proteomics. From three separate experiments, 21 proteins were reproducibly more than or equal to twofold decreased in CM from *Pdia3^−/−^* MEFs relative to WTCM and 7 proteins were more than or equal to twofold increased. Although not apparent by fluorescence microscopy or immunoblotting, PDIA3 was among the downregulated proteins (Supplemental Table S1; see https://doi.org/10.6084/m9.figshare.14852670). However, consistent with immunoblot data (Fig. 4), FN was ∼1.5-fold decreased and TSP1 was ∼1.5-fold increased in CM from *Pdia3^−/−^* MEFs relative to WTCM (Supplemental Table S1). Gene Set enrichment analysis of the identified gene products against the Molecular Signatures database identified the suite of proteins (analyzed either on the basis of the 21 proteins more than or equal to twofold decreased or the total list of 28 proteins) to be significantly enriched against the Gene Ontology (GO) category of “ECM” and related protein hallmark signatures including “matrisome,” “growth factor activity,” and “epithelial-mesenchymal transition” (EMT; Fig. 7*A*). Among the heparin-binding proteins of reduced abundance in the CM of *Pdia3^−/−^* MEFs, cell communication network 2 (CCN2; referred to in the older literature as connective tissue derived growth factor, CTGF, and having the alternative gene name *ctgf*) was notably fivefold decreased in the absence of PDIA3. CCN2 was also represented in nearly all of the enriched gene signature categories (Fig. 7*A*). In STRING analysis of protein function networks represented by the 28 proteins plus TSP1 and fibronectin, CCN2 (referred to in the STRING output as ctgf) was one of the most highly networked proteins (Fig. 7*B*). These results suggested a possible functional significance of CCN2 in the properties of WTCM.

### CCN2 Is a Key Factor for PDIA3-Dependent Cell Adhesion

**Figure 8.**
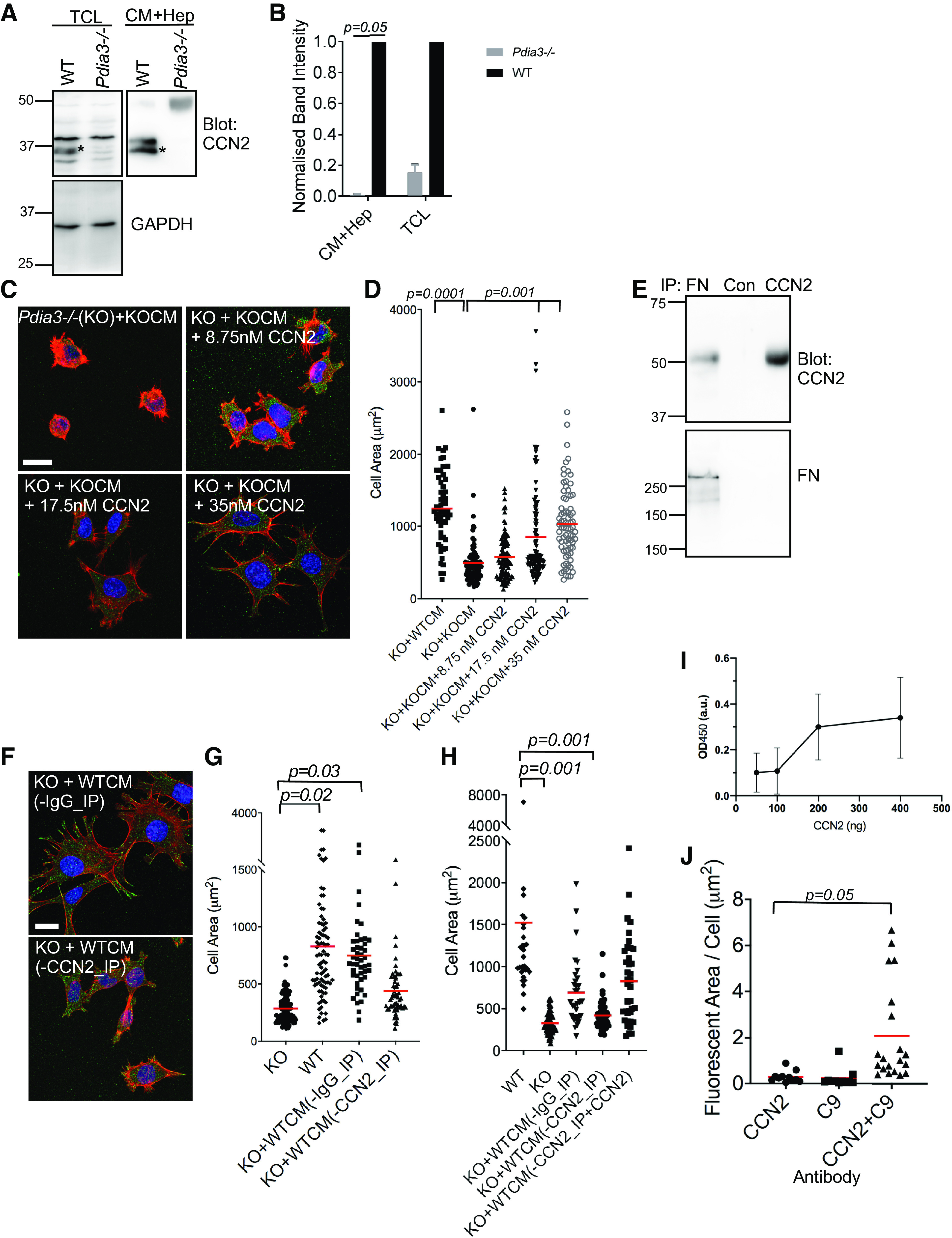
PDIA3 modulates cell adhesion through CCN2. *A*: representative immunoblots showing higher amounts of CCN2 protein in total cell lysate (TCL) and heparin-agarose pulldowns of CM (CM + Hep) from WT-MEFs than in equivalent samples from *Pdia3^−/−^* MEFs. GAPDH was used as a loading control for TCL. *B*: quantified analysis of CCN2 levels in TCL or heparin-agarose pulldowns of CM (CM + Hep) as in *A*. CCN2 bands (asterisks) from TCL were normalized against GAPDH band intensity. For each pair of samples, the WT sample was set as 1 and the *Pdia3^−/−^* sample normalized against this. Each column represents the mean and error bars the standard deviation. Analysis by paired *t* test. From *n* = 3 experiments. *C* and *D*: recombinant CCN2 confers adhesion-promoting activity on CM from *Pdia3^−/−^* MEFs. *C*: representative immunofluorescence images of F-actin and vinculin (the merged images also include DAPI stain) in *Pdia3^−/−^* MEFs (KO) plated for 2 h with CM from *Pdia3^−/−^* MEFs (KOCM), or KOCM plus the indicated concentrations of recombinant CCN2, examined by confocal microscopy and presented as XYZ stacks. Scale bar = 20 µm. *D*: quantified analysis of cell areas of *Pdia3^−/−^* MEFs (KO) under the conditions as indicated, from *n* = 3 experiments. *E*: immunoprecipitation of CCN2 or fibronectin (FN) from 48 h WTCM, followed by immunoblotting for CCN2 (*top*) or fibronectin (*bottom*). Nonimmune rabbit IgG (Con) was used as a negative control. *F* and *G*: immunodepletion of CCN2 from WTCM [WTCM(-CCN2_IP)] impairs its adhesion-promoting activity on *Pdia3^−/−^* MEFs. *F*: representative confocal immunofluorescence images for actin and vinculin (the merged images also include DAPI stain) in *Pdia3^−/−^* MEFs (KO) incubated with WTCM either after immunodepletion with a control IgG [WTCM(-IgG_IP)] or with anti-CCN2 [WTCM(-CCN2_IP)], presented as XYZ stacks. Scale bar = 20 µm. *G*: quantified analysis of cell areas of *Pdia3^−/−^* MEFs plated as in *F* under the indicated conditions for 2 h. From *n* = 3 experiments. *H*: add-back of CCN2 to immunodepleted WTCM [WTCM(-CCN2_IP+CCN2)] restores cell spreading of *Pdia3^−/−^* MEFs. Quantified analysis of cell areas of *Pdia3^−/−^* MEFs plated under the indicated conditions for 2 h. From *n* = 3 experiments. In *D*, *G*, and *H*, each datapoint represents one cell and red horizontal bars indicate the mean. Statistical analysis by ANOVA with Welch’s test. *I*: In vitro binding of TSP1 to surface-bound CCN2. Datapoints show means and error bars show standard deviation. *J*: quantitative analysis of fluorescent area per HDF cell by in situ proximity ligation with antibodies to CCN2 or TSP1 (C9) as indicated. Each datapoint is one cell and red horizontal bars indicate the mean. Analysis by Kruskal–Wallis test. From *n* = 3 experiments. CCN2, cell communication network 2; CM, conditioned medium; Con, control; KO, knockout; MEFs, mouse embryonic fibroblasts; WT, wild type; WTCM, conditioned medium from wild-type MEFs.

For the above reasons and because CCN2 is also a known promoter of cell adhesion (52), we selected CCN2 for further study as a possible PDIA3-dependent, adhesion-promoting, secreted protein. By immunoblot, CCN2 was validated to be decreased in *Pdia3^−/−^* MEFs relative to WT-MEFs, with reference to TCL and the heparin-affinity fraction of CM (Fig. 8*A*, and quantified from multiple immunoblot experiments in Fig. 8*B*). The presence of two bands of CCN2 in the samples may be due to a phosphorylated form at ∼44 kDa and the unmodified form at ∼36 kDa (53) or could possibly relate to glycosylation status (54). The higher molecular weight bands are likely to result from SDS-resistant oligomers of CCN2 or their cleavage products (55).

We then tested whether CCN2 alone confers adhesion-promoting activity on CM from *Pdia3^−/−^* MEFs, by adding recombinant CCN2 protein to CM from *Pdia3^−/−^* MEFs. A concentration-dependent increase in cell spreading was observed (Fig. 8*C* shows representative images, and quantification of cell areas from multiple experiments is shown in Fig. 8*D*). Using an antibody to CCN2, which immunoprecipitates CCN2 efficiently (Fig. 8*E*, *top*), we also found that WTCM immunodepleted for CCN2 was inactive to stimulate spreading and microfilament bundles in *Pdia3^−/−^* MEFs (Fig. 8*F* shows representative images, and quantification of cell areas from multiple experiments is shown in Fig. 8*G*), confirming a functional significance for CCN2 in WTCM. On add-back of recombinant CCN2 to WTCM immunodepleted of CCN2, the activity of WTCM to promote cell spreading was restored and equivalent to that of the WTCM control immunoprecipitated with nonimmune IgG (Fig. 8*H*). Thus, CCN2 has a necessary role in the adhesion-promoting activity of the CM of wild-type MEFs.

Our data (Fig. 1) identified TSP1 as a PDIA3 binding protein, and we tested whether CCN2 also binds to PDIA3 in vitro. Over CCN2 concentrations from 50 to 400 ng, no binding to PDIA3 was detected (data not shown). CCN2 is a known binding partner of fibronectin (Fig. 7*B*) (56) and we examined if CCN2 also binds TSP1. CCN2 was found to have concentration-dependent binding activity for TSP1 in vitro (Fig. 8*I*). We therefore examined whether TSP1 and CCN2 colocalize in cells by in situ proximity ligation in HDF cells. The signal (quantified as fluorescent area/cell) from HDF cells stained with antibodies to both CCN2 and TSP1 was significantly increased over the signals obtained with separate single antibody stainings (Fig. 8*J*), indicating close intracellular proximity of CCN2 and TSP1.

## DISCUSSION

By the method of BioID that identifies proximal interacting proteins, and substantiated by subcellular colocalization, in situ proximity ligation, and direct binding in vitro, we identify a novel, ER-located interaction between PDIA3 and the matricellular protein, TSP1. We build on this discovery to demonstrate that perturbation of PDIA3 function in fibroblasts (by two separate approaches, pharmacological inhibition and gene-knockout cells) alters properties of ECM and conditioned media (i.e., the secretome) in ways that have consequences for support of fibroblast adhesion. In investigating the molecular basis for this effect, we utilized comparative TMT-proteomics analysis of conditioned media from wild-type or *Pdia3^−/−^* MEFs and identified that PDIA3 promotes the extracellular abundance of a suite of heparin-binding proteins enriched for known ECM, “matrisome,” or epithelial-mesenchymal transition-associated roles. Of these proteins, we show that CCN2, but not fibronectin, has a necessary functional role in supporting fibroblast-ECM adhesion. The data support the concept that manipulation of PDIA3 activity may provide a new opportunity to manipulate the composition of ECM and, thereby, the local physiological microenvironment and cell behaviors. While this paper was in review, an interaction between TSP1 and the ER stress response mediator protein kinase R-like ER kinase (PERK) was published (57). In this study, overexpression of TSP1 in vivo, in cardiomyocytes, activated the PERK ER stress pathway. PERK was not identified in our TSP1 interactome proteomics, likely because of the use of a different cell type and/or the lack of an ER stress response in the experimental context.

PDIA3/ERp57 is a member of the PDI protein family, all of which are characterized by one or more thioredoxin domains with a CXXC active site (38, 58). In general, these domains allow reduction, isomerization and oxidation of disulfide bonds in newly synthesized proteins. The most well-studied family member is protein disulfide isomerase (PDI/PDIA1/P4HB) (58), which is known to interact with TSP1 extracellularly on the surface of platelets to catalyze the formation of a TSP1-thrombin-antithrombin III complex (59–61). PDIA3 and three other members of the PDI family (PDIA2/PDIp, PDIA4/ERp72, and PDILT) have domain architectures related to PDI, consisting of two catalytically active thioredoxin domains (a, a’) flanking two catalytically inactive thioredoxin domains (b, b’) (38). To date, a functional interaction between TSP1 and any of these four proteins has been unknown. In the ER, PDIA3 interacts with calreticulin and calnexin to act specifically in the posttranslational refolding of monoglycosylated glycoprotein substrates through reduction and isomerization of disulfide bonds (35, 36, 38, 62–64). PDIA3 also interacts with stromal interaction molecule 1 (STIM1) in the ER lumen to modulate its role in store-operated Ca^2+^ entry (SOC), such that SOC is enhanced in the absence of PDIA3 (65).

PDIA3 is an understudied member of the PDI family, yet it is expressed widely in mammalian tissues and several lines of research suggest particular roles in controlling proteins important for cell interactions with the microenvironment. Whereas *Pdia3* knockout in mice is embryonic lethal (27), *Pdia3^−/−^* MEFs or *Pdia3*-silenced MEFs show little alteration in ER structure, protein composition or function (27, 64, 66). However, STAT3 signaling is compromised (27). Specific knockout of PDIA3 in B cells inhibits recruitment of MHC class I molecules onto the peptide-loading complex for antigen presentation (67). PDIA3 is upregulated and secreted by kidney fibroblasts in response to TGFβ1 (34), is released on platelet activation (68), and its upregulation has been correlated with pulmonary fibrosis (69). Cell-surface PDIA3 has also been shown to act with glycosylated calnexin to reduce extracellular disulfide bonds and make ECM more susceptible to degradation by cancer cells (39). In breast cancer cells, PDIA3 activity was implicated in regulation of cell spreading and migration (37).

Most known substrates of PDIA3 depend on calreticulin and calnexin as cargo adaptors (63, 64). We establish here that TSP1 binds PDIA3 directly in vitro. All domains of TSPs contain disulfide bonds, with particular complexity in the type 3 repeats and C-terminal, L-lectin-like domain where a free thiol is distributed by intramolecular isomerization between twelve cysteine residues (70–72). We demonstrate that TSP1 protein is increased in *Pdia3^−/−^* MEFs or in HDF treated with the inhibitor 16F16. This suggests that, under normal conditions, refolding by PDIA3 may act as a brake on TSP1 secretion. Indeed, pulse-chase experiments have indicated that folding of each polypeptide of TSP1 normally continues after trimerization (73). The increased reactivity of antibody D4.6 with TSP1 secreted by 16F16-treated HDF is indicative of incorrect disulfide-bonding and partial unfolding, resulting in an aberrant diffuse patterning (and likely functional impairment) in the ECM. The diffuse ECM localization may be due to multiple factors, including the altered folding of TSP1 resulting in its altered binding to several proteins within the ECM, as well as the reduced amount of fibronectin as a binding partner within the ECM. A limitation of the use of 16F16 is that it is not specific to PDIA3 but also inhibits PDIA1 (26) and possibly PDIA6 (74). However, the observed congruence of phenotypic effects of 16F16 treatment with the properties of CM of *Pdia3^−/−^* MEFs gives confidence in the relevance to PDIA3 inhibition.

An important functional consequence of inhibition or loss of PDIA3 function at the cellular level is an alteration of cell attachment properties. Dihazi et al. (34) implicated qualitative changes in cell shape in the absence of PDIA3 by phase contrast microscopy. Our laboratory identified quantitative reductions in cell spreading in correlation with altered F-actin organization in human breast cancer cells treated with 16F16 (37). Here, we identify that fibroblasts undergo changes in cell area, F-actin and focal adhesion assembly on inhibition or gene knockout of PDIA3. Through conditioned media swap experiments we establish that these altered cell phenotypes are corrected by extracellular, heparin-binding proteins from WTCM. *Pdia3^−/−^* cells exposed to ECM isolated from either WT-MEFs or *Pdia3^−/−^* MEFs also show similar differences in cell adhesion.

Integrin β1 and several ECM proteins have been identified as PDIA3 substrates by a substrate-trapping approach; these include collagen α (VI), laminin β and γ chains and lysyl oxidase homolog 2 (62, 66). Immunoprecipitation of PDIA3 from a human renal fibroblast cell line under ER stress conditions identified fibronectin and collagen α-1(I) as coimmunoprecipitating ECM proteins (34). From our comparative quantitative proteomics of conditioned media from WT-MEFs or *Pdia3^−/−^* MEFs, we confirm here a dependence of extracellular collagens and LOXL2 on PDIA3, and further identify a suite of cysteine-rich, matrisomal proteins (including fibronectin, TSP1 and CCN2) to be dependent on PDIA3.

In view that fibronectin-depletion did not recapitulate effects of loss of PDIA3 function on WTCM properties, we prioritized CCN2 as a key protein for further analysis. CCN2 is a secreted matricellular glycoprotein of the CCN family that has several cell-surface receptors and numerous roles in vivo including cell adhesion, angiogenesis, and wound repair (75, 76). Furthermore, the four domains of CCN2 all include disulfide bonds (75). Our data demonstrate that CCN2 has a required role in the adhesion-promoting activity of the PDIA3-dependent secretome of fibroblasts. Depletion of CCN2 from WTCM strongly reduced its activity to promote *Pdia3^−/−^* MEFs cell spreading, and this effect was reversed by specific re-addition of recombinant CCN2. Also, CM from *Pdia3^−/−^* MEFs gained CCN2-concentration-dependent adhesion-promoting activity after addition of recombinant CCN2. There is currently very little information on mechanisms of chaperone regulation of CCN2 folding. Our experiments provide initial new data to suggest that, by its capacity to bind PDIA3 and CCN2, intracellular TSP1 might facilitate access of PDIA3 to CCN2. Future work will be needed to identify in general which of the proteins identified from the heparin-binding secretome interact directly with PDIA3 and which may be affected indirectly as a consequence of loss of PDIA3 function (for example, proteins trafficked as passengers through binding to PDIA3 substrates). It is plausible that CCN2 would have a central function role, as highlighted by its functional network connections with other PDIA3-dependent proteins from the secretome proteomics. CCN2 binds directly to fibronectin, and brings about modulation of chondrocyte adhesion (56) and to VEGF, leading to negative extracellular regulation of angiogenesis (77). Overall, our data support the concept that PDIA3 acts as a posttranslational nexus in the control of extracellular ECM production. Thus, manipulation of PDIA3 or other relevant resident proteins within the secretory pathway offers a way to leverage physiological changes in ECM quantity and/or quality. This may be an applicable strategy for tailoring cell microenvironments or cell-derived ECM with different functional properties.

## SUPPLEMENTAL DATA

10.6084/m9.figshare.14852670 Supplemental Table S1: https://doi.org/10.6084/m9.figshare.14852670.

10.6084/m9.figshare.14852661 Supplemental Figs. S1–S3: https://doi.org/10.6084/m9.figshare.14852661.

## GRANTS

This work was supported by MRC K018043. BBSRC/EPSRC L01386X supported the HyVolution imaging.

## DISCLOSURES

No conflicts of interest, financial or otherwise, are declared by the authors.

## AUTHOR CONTRIBUTIONS

A.L.H., K.J.H., and J.C.A. conceived and designed research; A.L.H., K.J.H., and M.A.J. performed experiments; A.L.H., K.J.H., M.A.J., and J.C.A. analyzed data; A.L.H., K.J.H., M.A.J., and J.C.A. interpreted results of experiments; A.L.H. and J.C.A. prepared figures; A.L.H., K.J.H., and J.C.A. drafted manuscript; A.L.H., K.J.H., M.A.J., and J.C.A. edited and revised manuscript; K.J.H., M.A.J., and J.C.A. approved final version of manuscript.
